# Marriage—Or What?

**Published:** 1888-09-01

**Authors:** 


### THE DOCTOR'S PULPIT.

### MARRIAGE—OR WHAT?

MRS. MONA CAIRD has gained what she probably most desired in writing her peculiar paper on the marriage question—viz., notoriety. We do not propose to add to that notoriety by a consideration of her sentiments and opinions, but to take up the question of marriage or no marriage from the medico-scientific point of view. Is marriage beneficial or harmful for the sexes? Is monogamy, on the whole, to be preferred to polygamy? A sober discussion of these two questions will not be without its value at the present time.

What do we mean by marriage? The lifelong union of one man with one woman, excluding all others. This is the almost universal rule in civilised and Christian communities. Is it a good rule; is it the best; does it carry within itself the elements of permanence? The coming together of the sexes in some kind of union is so universal as to be clearly and unmistakably a law of nature. It is a fact observed, not merely in the human species and among the higher vertebrates, but practically throughout the entire animal kingdom. Only in certain low forms of animal life the two sexes are to be found united in one and the same organism. The same is generally true of plants, as every student of elementary botany knows. The wild flowers of the woods and the most beautiful favourites of the garden and the conservatory are all made "male and female."

Men and women, in coming together in marriage, clearly do that which Nature intended they should do. Does Nature also point out in detail the lines and limits which should regulate the union of the sexes? Not so minutely as she points out that union itself is inevitable, but still with considerable clearness. There are certain broad limits both in the animal and plant worlds, outside which the union of the sexes never takes place. The hand of Nature points out those limits with a precision and definiteness which no man of science ever calls in question. Thus, and thus alone, is made possible the preservation of distinct species. Thus, and thus alone, is a confusion avoided which might result in forms the most horrible and grotesque, in scenes the most maddening and inconceivable. "Nature," or a "stream of tendency," or God—the last, as we think—has established a line of order, limits and bounds of demarcation as absolute as the towering cliffs that bid defiance to the sea. "Thus far shalt thou go, and no farther," is a law stamped by the iron hand of necessity on all things which come within the ken of man's experience.

This is an argument for limitations of some kind, and it is of the greatest possible significance. It is not, however, to be pressed too far. It precludes what seems to have been thought not very unnatural in ancient times—the union of man with the lower animals. Within the human species, man is left to the exercise of his voluntary choice. Science shows, as plainly as possible, the unity and homogeneity of the human race.

Experience precedes scientific generalisation, and combines with it in the elucidation of truth. An appeal to it shows that in every variety of human society love is a constant factor; love precedes and leads to marriage. We do not stop to define love. The fact is indisputable. At one period of life probably every normally constituted human being has loved one person, and has imagined that the full and perfect possession of that one person would make him or her completely and permanently happy. The feeling of the average man is not for women at large, but for one particular woman; and conversely, the feeling of the woman is for one particular man. Adam was happy with Eve, and if each of them had lived and remained as ideally perfect as the lover's imagination had declared them to be, no thought of any other marriage or association would have been possible. The same may be said with perfect truth of Antony and Cleopatra. There was a moment when each conceived the other to be the sum and essence of all perfections. Let but the perfections, imaginary though they were, have remained as an article of belief, and any other union than the one would have been undreamt of and impossible.

It is clear, then, that the typically perfect union of the sexes is the permanent marriage of one pair. How far is this typically perfect union observed in actual experience? In other words, What is the proportion of happy marriages? By happy marriages we do not mean those only in which the pair, as the result of temperament, live in a highly-idealised atmosphere of conscious mutual love. All marriages may be described as happy which bring to the married pair a reasonable amount of content and mutual satisfaction. The husband who thinks that on the whole his wife suits him fairly well; the wife that regards her husband as in the main a man to be trusted and cared for; the pair thus circumstanced are not to be pitied; their married life is in no sense a failure; they are reasonably happy.

What proportion of all the marriages contracted in civilised communities will come under this category? The writer has had the experience of a medical man in large practice for fifteen years, and this experience has included almost all orders of the population of this country, but has been specially among the middle and upper middle classes. It seems to him, as a candid observer, that at least 90 per cent. of ordinary marriages result happily. Even a higher percentage than this, say 97 or 98, would probably be nearer the truth.

But if this be so, must not marriage be considered to have reached as high an average of successful results as most other social arrangements? Must it not be considered to have reached a higher average than many? Human affairs are not largely happy in any but a very modified sense. Carlyle, and many other equally eminent writers, declare happiness to be comparatively a secondary affair. Very young, very immature, and very egotistical people no doubt regard happiness, and particularly their own happiness, as a question of the very supremest moment. But that is because they have not yet shed their milk-teeth. Probably, even in so short a time as ten years, if they live and associate with thinker so long, they will not be able to look back upon their present selves with any degree of patience.

A consideration more important than that of mere happiness is that of health of body and mind. What is the effect of marriage upon health, bodily and mental? There is not a medical man in the world who will not say that the healthiest of all conditions for adults of both sexes is marriage—other things being equal. This question of health alone is very broad, and will repay a separate consideration. It may be stated generally that to attain the highest perfection of bodily and mental health marriage is necessary for robust people of adult age. This is not the same as saying that marriage is necessary to health. Not only does marriage conduce to physical and mental well-being, but experience shows that ordinary monogamous marriages are at the top of the scale in this respect. From the standpoint of the scientific medical man, monogamous marriage, as it now prevails, gives the best results, in bodily and mental health to both sexes, which the world has yet seen.

A final question of first-rate importance may now be asked: What kind of union of the sexes produces the highest results in the physical, mental, and moral development of the race? To this there is only one answer which will command universal assent. That answer is, "Ordinary monogamous marriages." The experience of the whole civilised world is conclusive on this point. But what is the significance of the conclusion? It is this, that if thousands of years of experience have given such a positive demonstration that no sane person will question it, there is little likelihood that the egotistical and petulant discontent of one or two will bring about any considerable change. Many persons are dissatisfied with the prevalent social arrangements with regard to property, and show their dissatisfaction, not by writing to the magazines, but by helping themselves to their neighbour's goods. Probably if any magazine would open its pages to them, some of these deep-thinking philosophers would make out a very readable plea. This is but an exampleof innumerable similar cases. There are all sorts of causes of discontent in the world. How many women are unhappy because they have got red hair; how many because their noses turn up at the tip? What vast numbers of tailors think they would look well in the vicar's surplice, and how many vicars feel that they were born to wear lawn sleeves? It is too ridiculous that men and women should perturb themselves about so contemptible a trifle as a magazine article by a petulant and undisciplined woman. These three considerations alone—that the vast majority of marriages are reasonably happy; that they are conducive to the highest health of the married pair; that they produce the best results known to science in the physical, mental, and moral devevelopment of the race—will be regarded by most practical people as more than sufficient for the extinguishment of Mrs. Mona Caird's farthing rushlight.

